# Clear cell carcinoma arising in an ovarian remnant 19 years after oophoerctomy: case report

**DOI:** 10.1186/s12905-023-02695-4

**Published:** 2023-10-27

**Authors:** Ting-ting Yao, Shao-jie Zhao, Bing Zhang

**Affiliations:** Department of Gynaecology, Wuxi Maternity and Child Health Care Hospital, Affiliated Women’s Hospital of Jiangnan University, Jiangsu, 214002 China

**Keywords:** Ovarian remnant syndrome, Ovarian clear-cell carcinoma, Malignant transformation, Complete excision

## Abstract

**Background:**

Ovarian remnant syndrome (ORS) is a rare complication that occurs after oophorectomy, characterized by residual ovarian tissue causing pelvic pain, masses, and various symptoms. The clinical manifestations of ORS are nonspecific, and its diagnosis relies on histological examination. Since ORS typically represents a benign ovarian lesion, there have been few reported cases of malignant transformation. In this report, we presented a unique case of ovarian clear cell carcinoma (OCCC) arising from an ovarian remnant following salpingo-oophorectomy.

**Case presentation:**

Our patient was a 47-year-old female initially diagnosed with uterine myoma. She had previously undergone cesarean section and unilateral salpingo-oophorectomy. Transvaginal ultrasound and computed tomography (CT) scans revealed a soft tissue mass adjacent to the right lateral wall of the myometrium. The patient opted for transabdominal hysterectomy, left adnexal resection, laparoscopic omentectomy, appendectomy, and pelvic and abdominal lymphadenectomy. The final pathology results confirmed the diagnosis of OCCC, consistent with ORS. The patient subsequently received six cycles of intravenous chemotherapy using the carboplatin/paclitaxel (TC) regimen (paclitaxel liposomes 175 mg/m², carboplatin AUC 5). After 3 years of follow-up, the patient’s condition remained normal.

**Conclusion:**

ORS can significantly impact patients’ quality of life and pose challenges for clinicians. Complete excision of ovarian tissue during the initial surgery is crucial in preventing ORS recurrence and potential malignant transformation of ovarian remnants.

## Introduction

Ovarian remnant syndrome (ORS) is a rare complication following oophorectomy, characterized by residual ovarian tissue that leads to pelvic pain, masses, and other symptoms. The diagnosis of ORS relies on histological examination, typically in patients who have undergone previous oophorectomy [1]. Initially, the definition of ORS only included cases with bilateral oophorectomy, but it has since been expanded to unilateral oophorectomy cases whose residual ovarian tissue is found on the same side as the prior surgery [2]. Currently, there has been limited available data on the incidence of ORS, which primarily consists of case reports and retrospective case series studies. Malignant transformation of ORS is exceptionally rare. Here, we presented a case with malignant transformation from ORS to OCCC.

## Case presentation

**Fig. 1:**
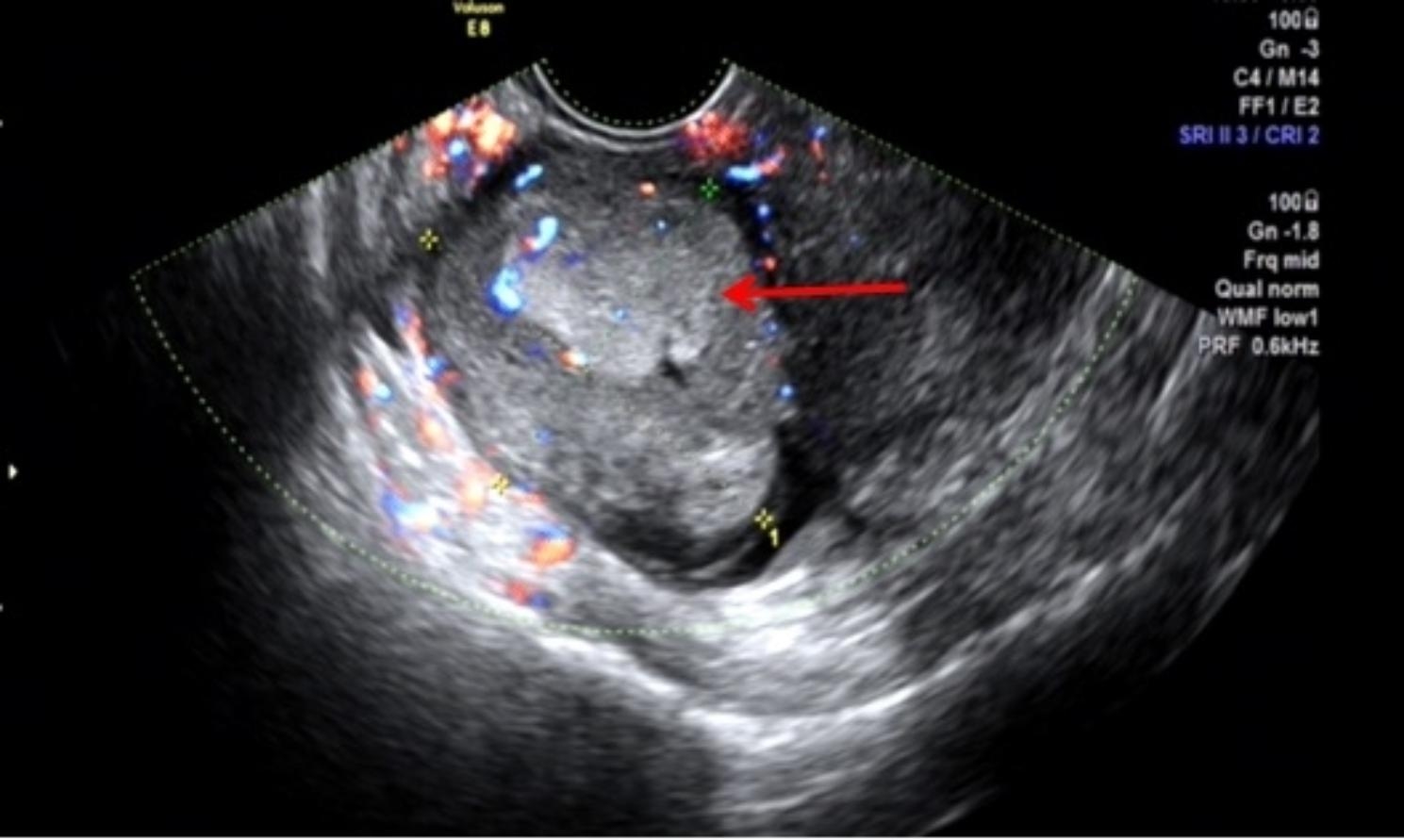
Transvaginal ultrasound showed an uneven high echo (red arrow) with a size was 48*50*39 mm in the right anterior myometrium

**Fig. 2:**
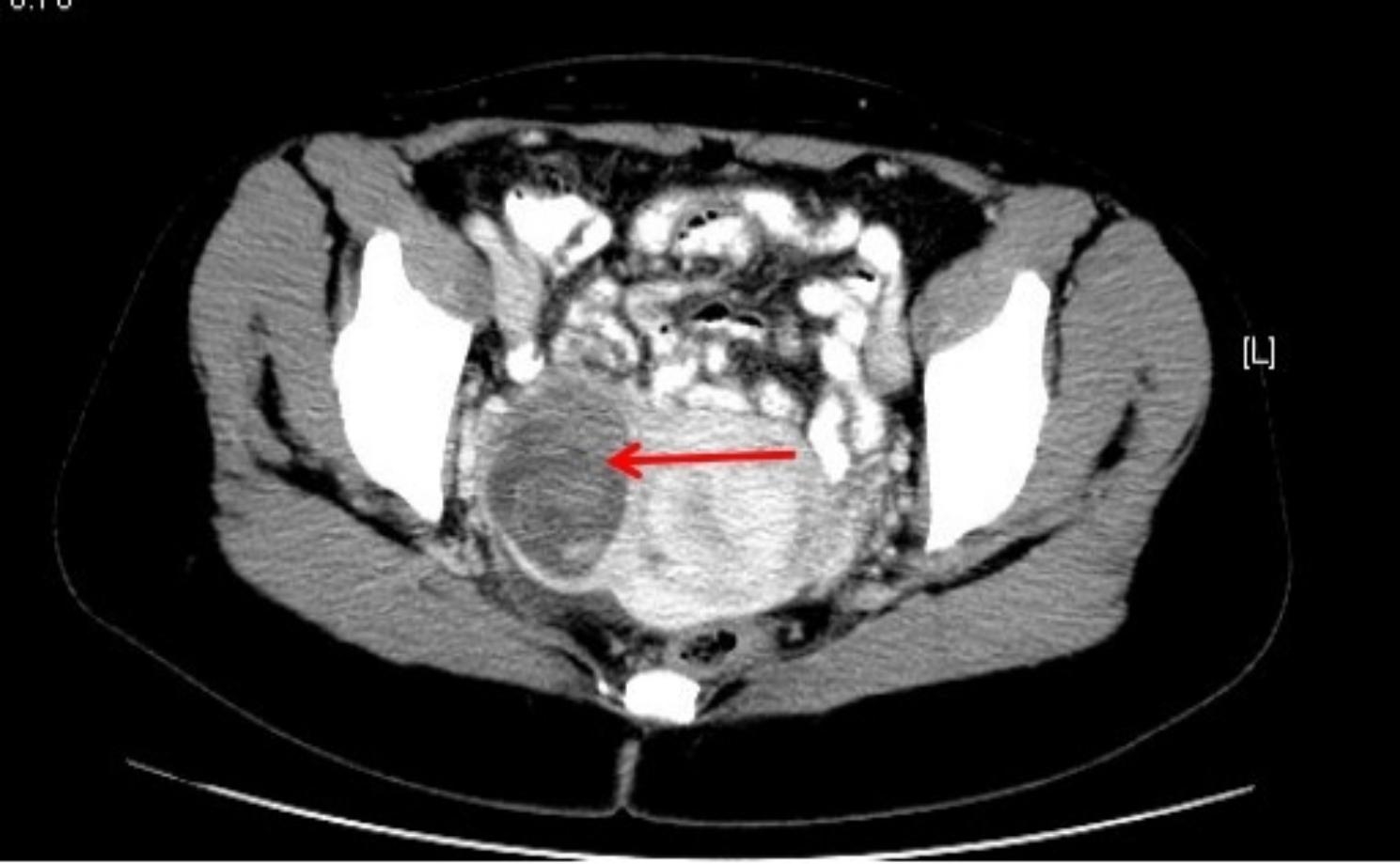
CT showed a round low-density soft tissue mass (red arrow) with a size of approximately 46*40 mm beside the right lateral wall of the myometrium

The patient, who complained of dull lower abdominal pain for the six months preceding her presentation, especially in the right side, was admitted to our hospital in 2020. The patient was a 47 years old woman accountant living in China. She reported experiencing G2P1A0 and does not give history of use of contraception. The patient had previously undergone a lower segment cesarean section and unilateral salpingo-oophorectomy due to umbilical cord entanglement during childbirth 19 years ago. No other history beyond the cesarean was noted, and no history of any gynecological malignancy in the family. The general physical examination detected no abnormalities. The gynecological examination found a tumor mass located on the right posterior uterine wall, of 40 × 50 mm size. Transvaginal ultrasound (Fig. 1) showed a low echogenicity area measuring 9 × 10 mm in the posterior wall of the myometrium, an isoechoic area measuring 24 × 18 mm in the left wall of the myometrium, as well as heterogeneous hyperechogenicity measuring 48 × 50 mm in the anterior myometrium. Abdominal enhanced computer tomography (CT) (Fig. 2) revealed a rounded soft tissue mass approximately 46 × 40 mm in size within the right wall of the myometrium, displaying clear boundaries and lower enhancement density compared to the myometrium. Additionally, multiple lymph nodes adjacent to the right iliac vessels were detected, with the largest measuring 9 × 5 mm. Routine blood tests were taken. The results revealed: blood morphology - Hct 40.5%, Hb 12.8 g/100, RBC 4.37 T/L, WBC 7.59 G/L; glucose 4.38 mmol/L, urea 5.09 mmol/L, creatinine 57.5 umol/L, total bilirubin 12.3 µmol/L, diastase 60.7 U/L, AST 13.3 U/L, ALT 9.2 U/L, Na^+^ 139.5 mmol/L, K^+^ 3.86 mmol/L, FBG 4.76 g/L, CA125 181.4 U/mL, HE4 55.6 pmol/L, CA199 15.9 U/mL, CA153 10.6U/mL, CA72-4 3.5 U/mL, CEA 1.93 ng/mL, AFP 2.7 ng/mL, SCC 1.5 ng/mL. Furthermore, the patient had a history of uterine myoma for over a decade.

**Fig. 3:**
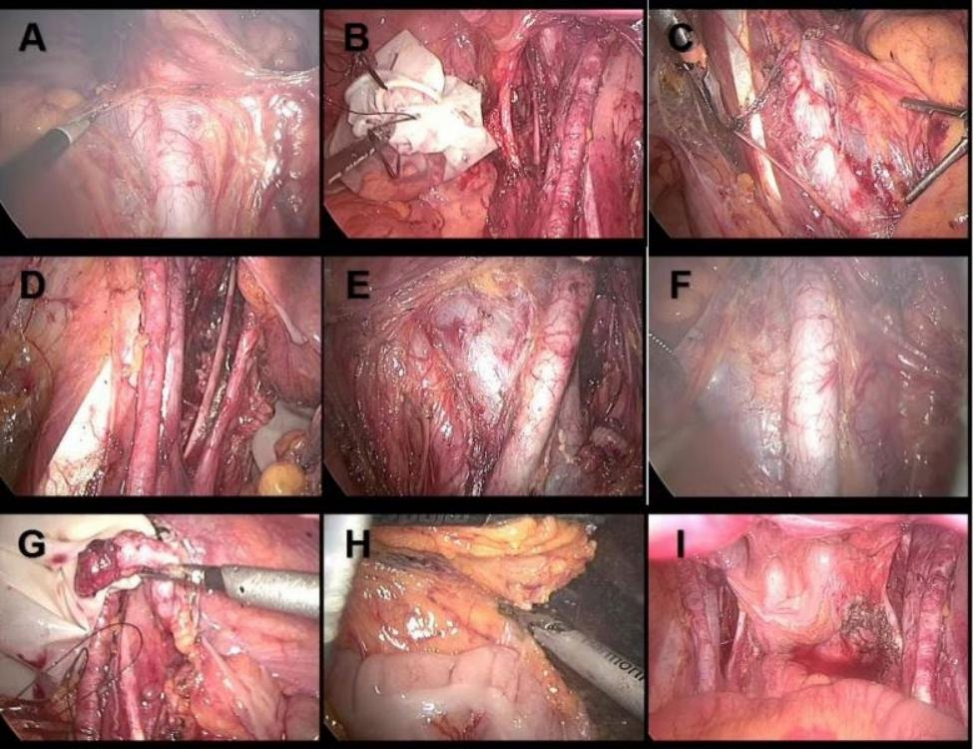
Laparoscopic surgical Atlas.View of right common iliac lymph node dissection (**A**) and right pelvic lymphadenectomy (**B**).View of left common iliac lymph node dissection (**C**) and left pelvic lymphadenectomy (**D**).View of presacral lymphadenectomy (**E**) and lymph node resection near abdominal aorta (**F**).View of appendectomy (**G**) and omentectomy (**H**). Postoperative view of pelvic cavity (**I**)

**Fig. 4:**
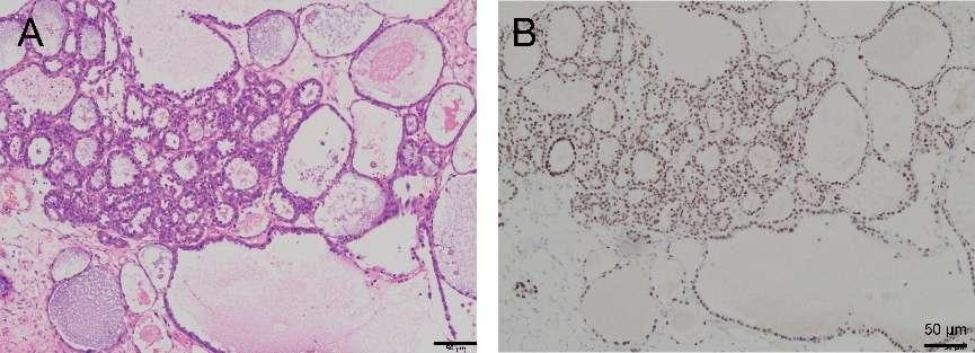
HE staining showed tumor cells had vacuolated, clear cytoplasm and basally located pleomorphic nuclei without prominent nucleoli, numerous tumor cells with clear cytoplasm, a key feature of OCCC (**A**). IHC demonstrated that tumor cells strongly expressed HNF1-βin a cytoplasm pattern (**B**)

Based on the patient’s medical history and imaging examination, a diagnosis of uterine leiomyoma was initially considered. Differential diagnoses should also be considered, such as uterine sarcoma and adenomyoma. However, a transabdominal hysterectomy with left adnexectomy was proposed after careful evaluation. The surgical approach utilized was a transverse incision from the previous operation. On the serosa of the uterus in the right accessory area, a pale-yellow mass measuring approximately 50 × 40 × 30 mm with a nodular appearance was discovered. The frozen section analysis reported clear cell carcinoma (CCC). Following urgent discussions with the patient’s family, the operative procedure was modified to include transabdominal hysterectomy, left adnexal resection, laparoscopic omentectomy, appendectomy, and pelvic and abdominal lymphadenectomy. Laparoscopic surgical Atlas is presented in Fig. 3. Hematoxylin-eosin (HE) staining in Fig. 4 displayed tumor cells with vacuolated, clear cytoplasm and pleomorphic nuclei located basally without prominent nucleoli, suggesting ovarian CCC (OCCC). Immunohistochemistry (IHC) demonstrated strong cytoplasmic expression of HNF1-β in tumor cells. Histopathology of paraffin blocks confirmed the presence of CCC on the serosa of the uterus in the right adnexal area, along with uterine leiomyoma. No abnormality was found in the left ovary. Metastatic carcinoma was observed in one out of five para-aortic lymph nodes, while anterior sacral, bilateral common iliac and pelvic lymph nodes were free from metastatic carcinoma. Moreover, peritoneal biopsy results of the right pelvic wall, upper abdominal wall, bilateral pelvic infundibulum ligament, appendix, omentum, and peritoneal lavage were negative for malignant cells. According to the International Federation of Gynecology and Obstetrics (FIGO) guidelines, the patient was diagnosed with stage IIIA1i OCCC. Genetic testing indicated normal breast cancer (BRCA) status and a homologous recombination repair deficiency (HRD) score of less than 1. The patient underwent six cycles of intravenous chemotherapy using the carboplatin/paclitaxel (TC) regimen (paclitaxel liposomes 175 mg/m², carboplatin AUC 5). After the second round of chemotherapy, CA125 levels returned to normal. Following completion of the sixth chemotherapy cycle, CT imaging revealed no abnormal pelvic masses or metastatic lesions. At the 3-year follow-up, the patient’s condition remains normal.

## Discussion

The incidence of ORS is low, and the resulting masses are typically benign, consisting of proliferating ovarian tissue or simple cysts. Symptoms of ORS can include chronic pelvic pain, pelvic mass, and urinary obstruction, caused by the growth and compression of remnant ovarian tissue. In some cases, residual ovarian tissue can continue to function, secreting estrogen and maintaining menstruation. This raises concerns when estrogen levels remain unchanged or when menopausal symptoms persist after bilateral ovariectomy. Studies [3]have shown that replantation of ovarian specimens in the abdominal cavity can be viable even without blood supply in a cat model. Ectopic implantation of ovarian tissue has also been reported in cases of ORS after laparoscopic ovarian cyst excision, resulting in abdominal incision and intestinal obstruction [4–6]. Although rare, malignant tumors can develop from ovarian remnants, including mucinous cystadenocarcinoma, endometrioid adenocarcinoma, and borderline serous neoplasia [7–11], suggesting the possibility of malignant transformation of remnant ovarian tissue. However, early clinical manifestations of ovarian cancer are often atypical, leading to advanced-stage diagnosis with a poor prognosis.

Misdiagnosis of ORS can occur for various reasons. One probable cause is the lack of recognition of ORS, especially in patients with a history of oophorectomy. Surgeons may not fully realize the need for complete removal of ovarian tissue and the potential for replantation and subsequent growth or malignant transformation. Early diagnosis and differential diagnosis of ORS are challenging, highlighting the importance of raising awareness about the disease. Ovarian endometriosis is associated with ovarian carcinoma in 50% of reported ORS cases [12]. In this particular case, it is suspected that the remaining ovary developed ovarian endometriosis, leading to chronic pelvic pain, pelvic mass, and eventual transformation into CCC.

To prevent the occurrence of ORS, careful evaluation of the feasibility of complete oophorectomy is necessary before the procedure. Factors such as a history of endometriosis and previous pelvic and abdominal surgeries can cause pelvic adhesions, making complete removal of ovarian tissue more difficult. In cases where oophorectomy is performed concurrently with a cesarean section, the surgeon should carefully consider the challenges associated with complete ovary removal, especially when the enlarged uterus obstructs visibility in the surgical field. Therefore, the decision to perform oophorectomy at the time of cesarean section should be made after careful consideration.

During surgery, meticulous and professional operation techniques are crucial to prevent the occurrence of ORS. The use of surgical skills, such as high ligation of the pelvic infundibulum ligaments, retroperitoneal dissection, and ovarian dissection [7]., has been demonstrated in a video released by the Canadian Academic Medical Center. Additionally, preventing ectopic ovarian tissue implantation can be facilitated by placing the ovary in a specimen bag before initiating surgery.

After oophorectomy, regular annual follow-up is essential to monitor any anomalies in the adnexal area. If any abnormalities are detected through transvaginal ultrasound, it is important to differentiate them from ORS. ORS poses a risk of malignant transformation, and early detection of ovarian cancer is challenging. Therefore, clinical manifestations, regular physical examinations, and tumor marker monitoring are crucial for the timely detection of ovarian cancer.

In this case, we simply ruled out ovarian involvement based on the patient’s history of right-sided salpingo-oophorectomy and uterine myoma. However, frozen section analysis revealed CCC, leading to an expanded surgical approach following the National Comprehensive Cancer Network (NCCN) guidelines. The surgical procedure involved transabdominal hysterectomy, appendix removal, greater omentum resection, and pelvic lymph node dissection. The original incision was along the patient’s previous transverse incision of cesarean section. Limited by the poor ductility of the transverse incision, the decision to use laparoscopy for omentectomy, appendectomy, and lymph node dissection was made after considering the aesthetic concerns of the patient and obtaining consent from her family. Postoperative recovery was successful, but it is important to avoid such complex surgical procedures in the future. The right adnexal resection during the previous cesarean section significantly increased the difficulty of completely removing ovarian tissue. Genetic testing indicated normal BRCA status and negative homologous recombination repair (HRR), leading to the administration of six cycles of intravenous chemotherapy using the TC regimen. Strict postoperative follow-up is crucial for such patients. If abnormalities are observed in the ipsilateral adnexa, ORS should be ruled out first.

## Conclusion

ORS can severely impact patients’ lives and present challenges for clinicians. This case highlights the possibility that a pelvic mass may originate from remnant ovarian tissue after unilateral or bilateral salpingo-oophorectomies. Preoperative frozen section assessment is essential to exclude malignancy. Complete excision of ovarian tissue during the initial surgery prevents the recurrence of ORS and potential malignant transformation.

## Data Availability

The datasets used or analysed during the current study available from the corresponding author on reasonable request.

## Abbreviations

ORS: Ovarian remnant syndrome
TC: Carboplatin/paclitaxel
CT: Computer tomography
CA-125: Carbohydrate antigen 125
HE: Hematoxylin-eosin staining
OCCC: Ovarian clear cell carcinoma
IHC: Immunohistochemistry
HNF1-β: Hepatocyte nuclear factor 1-β
FIGO: International Federation of Gynecology and Obstetrics
BRCA: Breast cancer
NCCN: National Comprehensive Cancer Network
HRR: Homologous recombination repair
